# Overview of the testing and assessment of effects of microbial pesticides on bees: strengths, challenges and perspectives

**DOI:** 10.1007/s13592-021-00900-7

**Published:** 2021

**Authors:** Shannon Borges, Abdulrahim T. Alkassab, Elizabeth Collison, Silvia Hinarejos, Ben Jones, Emily McVey, Ivo Roessink, Thomas Steeger, Maryam Sultan, Jacoba Wassenberg

**Affiliations:** 1 Office of Pesticide Programs, U.S. Environmental Protection Agency, Washington, DC, USA; 2 Federal Research Centre for Cultivated Plants, Institute for Bee Protection, Julius Kühn-Institut (JKI), Braunschweig, Germany; 3 Staphyt Ltd, Wetherby, UK; 4 Sumitomo Chemical, Saint Didier au Mont d’Or, France; 5 Fera Science Ltd, York, UK; 6 Dutch Board for the Authorisation of Plant Protection Products and Biocides (Ctgb), Ede, The Netherlands; 7 Wageningen Environmental Research, Wageningen, The Netherlands; 8 Bayer AG Crop Science Division, Monheim, Germany

**Keywords:** *Apis mellifera*, biopesticides, biological control, pathogenicity, toxicity

## Abstract

Currently, there is a growing interest in developing biopesticides and increasing their share in the plant protection market as sustainable tools in integrated pest management (IPM). Therefore, it is important that regulatory requirements are consistent and thorough in consideration of biopesticides’ unique properties. While microbial pesticides generally have a lower risk profile, they present special challenges in non-target organism testing and risk assessment since, in contrast to chemical pesticides, their modes of action include infectivity and pathogenicity rather than toxicity alone. For this reason, non-target organism testing guidelines designed for conventional chemical pesticides are not necessarily directly applicable to microbial pesticides. Many stakeholders have recognised the need for improvements in the guidance available for testing microbial pesticides with honey bees, particularly given the increasing interest in development and registration of microbial pesticides and concerns over risks to pollinators. This paper provides an overview of the challenges with testing and assessment of the effects of microbial pesticides on honey bees (*Apis mellifera*), which have served as a surrogate for both *Apis* and non-*Apis* bees, and provides a foundation toward developing improved testing methods.

## 1. INTRODUCTION

In general, the term “biopesticide” is intended to represent pesticides that are derived from natural materials (e.g. animals, plants, bacteria, minerals). Amongst regulatory agencies biopesticides normally fall into the following classes: biochemicals including natural products (e.g. plant extracts, minerals) and semiochemicals (e.g. pheromones), macroorganisms (e.g. insects, nematodes), products of biotechnology (e.g. plant incorporated protectants) and microbial pesticides (e.g. bacteria, fungi).

The market for biopesticides is reportedly growing at an average annual rate of 15% since 2010 (Marrone 2014; Pelaez and Mizukawa 2017; Damalas and Koutroubas 2018). Although this is less than the historic growth of the conventional synthetic chemical pesticide market, various factors have led many companies to devote resources to the development of biopesticides. Currently, biopesticides make up about 5% of the global plant protection market, with a current value of about US$3 billion worldwide (Marrone 2014; Olson 2015; Damalas and Koutroubas 2018) that is estimated to grow to US$4 billion by 2024 (Shukla et al. 2019). There are about 1400 biopesticide products representing about 1000 active ingredients sold worldwide (Meyer 2013; Balog et al. 2017), with approximately 400 biopesticide active ingredients in products registered in the United States of America (USA) (USEPA 2018) compared to about 68 active ingredients of biopesticides in the European Union (EU) (Meyer 2013).

The economic development of this sector can be related to several drivers: (1) The world population is now estimated to be 7.7 billion, and is expected to reach 9.7 billion in 2050 and 10.9 billion in 2100 (United Nations 2019); (2) global climate change and the increasing yield losses associated with different abiotic stressors (e.g. drought); (3) development of pathogen and pest resistance to conventional chemical plant protection products (i.e. pesticides) as well as a decline in the rate of discovery, development and registration of new chemical active ingredients with new modes of action; (4) the societal and regulatory pressures to reduce the pesticide residues in food and the environment; and, (5) the increased role of integrated pest management (IPM) in several countries (Pelaez and Mizukawa 2017; Sessitsch et al. 2018). Altogether and due to the limits in availability of arable land, it is reasonable to assess how to increase yield within the same footprint, (i.e. without substantially altering ecosystems and protecting environmental resources for future generations). Thus, developing effective and specific biopesticides, as well as adopting strategies in agricultural systems to be resilient to challenges associated with balancing sustainable food production with healthy ecosystems, have become drivers for both governments and industry alike (Ghini et al. 2012; Verger and Roobis 2013; Balog et al. 2017).

Out of all the approved biopesticides, microbial pesticides comprise the largest group (Shukla et al. 2019). Here, microbial pesticides include any microorganisms (i.e. bacteria, fungi, viruses, protozoans) which can be used for plant protection. A wide range of microbial pesticides has been developed during the last decades, with new species and strains of microorganisms frequently discovered as a result of this development effort (Köhl et al. 2019). According to Kabaluk and Gazdik (2007), in 2007, there were 225 microbial pesticide products available in countries affiliated with the Organisation for Economic Co-operation and Development (OECD). It was reported that about 175 microbial pesticide active substances are available and can be used in agricultural systems (Singh 2014; Arora 2019). Frederiks and Wesseler (2019) reported that 47 microbial pesticide active substances have been registered in the EU and 73 in the USA.

With the growing interest in developing microbial pesticides, and their increasing share in the plant protection market, it is important that regulatory requirements are consistent and thorough in consideration of the unique properties of microbial pesticides. A typical ecological risk assessment framework requires the evaluation of toxicity to non-target organisms in various environmental compartments and, in combination with predicted exposure, assessment of potential risks. Where biochemical pesticides (e.g. plant extracts) may be tested using established guidelines and protocols developed for conventional synthetic pesticides, hazard testing for microbial pesticides presents distinct challenges. For example, microorganisms are living organisms and are not readily “soluble” in the traditional sense in an aquatic exposure system. Furthermore, the conditions (e.g. temperature, pH, humidity, light) specified in the available chemical pesticide guidelines may not be conducive for microbe survival. Also, for microbial pesticides, it is not sufficient to test toxicity alone; the infectiveness and pathogenicity must be evaluated as well. Infectiveness (or infectivity) refers to the ability of a microorganism to enter a host and multiply within that host, whereas pathogenicity refers to the ability to cause disease (i.e. harm) to the host. As such, it is possible for a microorganism to be infective without being pathogenic, whereas to be pathogenic, it must also be infective.

In a similar manner to conventional chemical pesticides, the end-use biopesticide product applied in the field will typically be a formulation comprising the active substance/ingredient, as well as other co-formulants to give the product the necessary properties for handling, application and storage. In the case of microbial pesticides, while the active substance/ingredient may be defined as a particular strain (e.g. bacterium or fungus), it is important to recognise that the technical grade material, which is often used in non-target organism testing, is usually not a pure living organism, but may include also a mixture of spent fermentation media, metabolites or toxins produced by the microorganism and dead material.

## 2. AIMS OF THIS OVERVIEW

Because of the recognition that pesticides may be contributing to declines in some pollinator species (vanEngelsdorp et al. 2009; Pettis and Delaplane 2010; USDA 2013) and in response to the need for reliable data on which regulatory authorities can evaluate the potential for a pesticide to adversely affect non-target organisms (e.g. bees), this paper identifies the strengths and weakness in current testing methods and discusses opportunities for additional method development. Several aims of the overview were identified including:

- Identifying the current knowledge gaps related to testing with microbial pesticides and bees
- Identifying and addressing limitations of the current test guidelines / guidance regarding microbial pesticides
- Providing insights and future steps necessary to improve testing and risk assessment of microbial pesticides to bees

## 3. BRIEF OVERVIEW OF CURRENT REGULATORY RISK ASSESSMENT FRAMEWORKS IN THE USA AND EU, WITH REGARD TO MICROBIAL PESTICIDES AND BEES

The US and EU approaches are provided below only as examples of general approaches in two major agricultural markets. For a more detailed comparison of the US and EU approaches, see Frederiks and Wesseler (2019).

### 3.1. USA

In the USA, there are multiple statutes prescribing the regulation of pesticides, and the Environmental Protection Agency (EPA) is the lead federal agency responsible for regulating pesticides that are sold or distributed in the USA. While there are multiple statutes regarding the regulation of pesticides, the Federal Insecticide, Fungicide, and Rodenticide Act (FIFRA) is the primary federal law providing the EPA with the authority to grant a license (registration) to sell and distribute a pesticide. Under FIFRA, registrations must meet the FIFRA regulatory standard, i.e. the use does not result in unreasonable adverse effects to human health or the environment. FIFRA also specifies the data required to support registration; however, the EPA has flexibility in determining data requirements and can modify requirements on a case-by-case basis to more fully characterise the effects of a pesticide product. In determining whether a pesticide causes “unreasonable adverse effects” on the environment must under FIFRA take into account the economic, social and environmental costs and benefits of the use in which the agency balances risks versus benefits (USEPA 1990). However, the EPA recognises that certain conventional and microbial pesticides pose a lower risk to human health and the environment than existing alternatives and, as a result, the agency has developed processes to encourage the development of these reduced risk products and to more rapidly register commercially viable alternatives. Since biopesticides are naturally occuring, do not tend to persist and have a history of exposure for both humans and wildlife demonstrating minimal toxicity (Leahy et al. 2014), these products are frequently identified as representing less risk than conventional pesticides. Although these products must still meet the FIFRA standard, the EPA has the flexibility to streamline both the review process and the data used to inform that process.

Specific data requirements are set out for microbial pesticides, specified in the US Code of Federal Regulations (CFR) in Data Requirements for Registration (40 CFR Part 158, Subpart V), and specific microbial pesticide test guidelines are available under Office of Chemical Safety and Pollution Prevention (OCSPP) series 885. Data requirements have a tiered structure, in which tier I testing is required, but testing at higher tiers is only necessary when adverse effects are observed at lower tiers. With respect to effects on bees, the following data requirements apply in this tiered approach (USEPA 1996a, b; USEPA 2012):

- At tier I, OCSPP 885.4380 (honey bee testing)
- At tier II, OCSPP 885.5200 (terrestrial environmental expression tests)
- At tier IV, OCSPP 850.3040 (field testing for pollinators)

Tier I data are required on the active ingredient (active substance), whereas tier II data may be generated on either the active ingredient or end use product while tier IV data are required on the typical end-use product. Data routinely required under Part 158 may not always be sufficient to assess whether there are unreasonable adverse effects on the environment. Therefore, the EPA has flexibility under 40 CFR Part 158.30(b) and 40 CFR Part 158.75 to require additional data when needed to fully characterise the effects of a pesticide and to modify data requirements on a case-by-case basis.

### 3.2. EU

The approval and authorisation of microbial pesticide active substances and plant protection products in the EU fall under the same regulation as conventional chemical pesticides (i.e. Regulation (EC) No 1107/2009 (EC 2009a)). In general, the approval of microbial active substances is done at the strain/isolate level, with the exception of a family of DNA viruses (i.e. baculoviruses) which have been approved at the species level.

In this regulation, there are no specific executive considerations for any biopesticides including microorganisms. Nevertheless, a possible categorisation of an active substance or product as “low risk” is indicated, which includes several of the microorganisms currently approved in the EU. This enables a reduction of evaluation timelines, an increased duration of the authorisation period and a reduction in fees associated with the evaluation procedure.

Furthermore, the EU has developed a *Directive on Sustainable Use of Chemical Pesticides* (EC 2009b) (SUD) that aims to enhance the use of non-chemical alternatives to chemical pesticides. Waivers are recommended in cases of negligible or minimal exposure, or non-entomopathogenic agents, if data are available to support that claim.

On the other hand, data requirements for approval of active substances (Commission Regulation (EU) No 283/2013; (EC 2013a)) as well as plant protection products (Commission Regulation (EU) No 284/2013; (EC 2013b)) include a specific section (Part B) regarding microorganisms including viruses. This regulation indicates that data regarding effects on bees must include information on toxicity, infectiveness and pathogenicity to bees. Furthermore, unless justification can be provided to show that exposure of bees is unlikely, the regulation requires similar information to be reported for plant protection products containing microorganisms, where product-specific effects cannot be predicted from data on the microorganism. The regulation also specifies under data point 8 (vii) that the risk of relevant metabolites must be addressed: “it may be necessary to conduct separate studies for relevant metabolites (especially toxins), where these products constitute a relevant risk to non-target organisms and where their effects cannot be evaluated by the available results relating to the microorganism […]”.

To date, specific EU testing guidelines are not available for microbial pesticides and hence (as specified in Commission Regulation (EU) No 283/2013 (EC 2013a)) other available test guidelines (e.g. EPA test guidelines) or adapted OECD study designs are required to ensure these data requirements are fulfilled. Furthermore, although there are no guidance documents specific to the evaluation of microbial pesticides in the EU, the OECD Guidance on the environmental safety evaluation of microbial biocontrol agents (OECD Series on Pesticides No. 67; OECD 2012) is generally followed.

## 4. KNOWLEDGE GAPS IN RISK ASSESSMENT FOR MICROBIAL PESTICIDES

### 4.1. Hazard Assessment

In assessing risk, the tools used to inform regulatory decision-making should be fit-for-purpose (i.e. they must include endpoints which are biologically relevant and of regulatory importance, such as adult survival or sublethal effects that may essentially cause a reduction in survival) and provide reproducible, relatively selective (i.e. reasonable low number of false positives) and sensitive (i.e. capable of detecting effects at environmentally relevant exposure levels) results. Ideally, the methods used should be suitably evaluated across a wide range of labs as providing reproducible and consistent results and have curated reference chemicals with which to evaluate test performance. Tiered testing also affords the ability to test at multiple levels of complexity, so lower-tier studies can be more simplified whereas higher-tier studies can be more reflective of real-life exposure scenarios and include observation of more complex endpoints, such as behaviour. Multiple insect pollinator guidance documents and test guidelines have been developed to support regulatory decisions (Table I).

Some of the issues related to these test guidelines which have arisen relative to testing microbial pesticides are described in Table II.

These study design issues may give rise to generic limitations that require further investigation as noted in Table II. For example, at this time, little is understood about how well microorganisms survive in various test matrices in laboratory feeding studies. Microorganisms may or may not survive, or survival may vary between different types of microorganisms (e.g. bacteria versus fungi) or by genera or species. Further research is needed to determine the ability of many of these guidelines to evaluate exposure and effects to inform risk assessments. Thus, Table III identifies specific limitations that are known about each guideline that may need examination or correction to produce reliable and useful results.

Some knowledge gaps also exist in relation to the specific needs of bee testing with microbial pesticides and appropriate methods for testing. For example, the EPA’s OCSPP 885.4380 guideline is very general and does not provide sufficient detail as to the actual conduct of the study nor are criteria developed for gauging acceptability of the study. The OECD guidelines are focussed on a dose–response design to calculate a toxicity endpoint (e.g. LD_50_ value), but do not address methods to assess the presence or absence of pathogenicity. Therefore, studies conducted according to these guidelines may vary in experimental approach (e.g. test concentration, exposure duration, study duration, and types of controls), which can influence the reliability and consistency of results.

### 4.2. Exposure assessment

Knowledge gaps also exist in the assessment of exposure to bees, which affects the accuracy of risk estimates, as well as the determination of the proper exposure level in bee testing. Addressing knowledge gaps in our understanding of bee exposure in the field will improve the reliability and results of bee testing and risk assessment.

According to OECD No. 67, in general, the exposure of bees due to indoor and outdoor applications of microbial pesticides (e.g. spray application, granules, seed treatment) should be considered in the risk assessment. Risk assessments should be based on the specific microorganisms considering the intended use (soil or foliar applications), target pest (e.g. fungi, insect) and mode of action (pathogen, competition for space and nutrients, etc.). Argumentation to grant a waiver of the data requirement (i.e. a justification based on relevant scientific peer-reviewed open literature) is an option in case of (1) negligible or minimal exposure to bees (e.g. emissions due to spray drift from permanent greenhouse structures via open windows and openings can be considered negligible) and (2) in case of non-entomopathogenic microbial biological control agents (mBCAs), if database searches find no reports of detrimental impacts of the considered microorganisms on bees and other closely related species of the mBCA that share the same ecological habitat. The acceptance of these recommendations provided by the OECD 67 will depend on each regulatory agency.

Different methods are available to measure environmental concentrations of microbes at any given location or point in time such as plating, baiting, immunological techniques and DNA-based techniques including real-time PCR and next-generation sequencing techniques (for further details see Köhl et al. 2019). However, models for the determination of the estimated environmental concentration (EEC)/predicted environmental concentration (PEC) in nectar and pollen for microbial pesticides do not yet exist. Such models would provide risk assessors with the ability to estimate the dynamics of microbial population size. Fungal and bacterial strains may be considered to have the potential to survive and to become established in the environment under certain conditions, and even to increase in nectar and pollen because of the presence of nutrients in pollen and nectar, necessary for vegetative growth of the microorganisms. However, many factors, including intrinsic factors (e.g. intrinsic stability and viability of fungal propagules, bacterial spores and their vegetative cells), abiotic factors (e.g. survival of certain microorganisms possible only under a restricted range of pH, temperature and/or humidity conditions; UV light strongly affecting persistence of most microorganisms) and especially biotic factors (e.g. competition for space and nutrients with natural occurring microorganisms), constrain the survival and persistence of microorganisms in the environment (Scheepmaker and Butt 2010; Köhl et al. 2019). Viruses, instead, including baculoviruses and resistance inducing plant viruses, are highly specific to their host, and not able to replicate outside their host. Considering the differences in the mode of action, life cycle, survival and interactions with other living organisms (both macro- and microorganisms), the probability of developing a single model adequate for the determination of the environmental concentration of a microorganism is very low. Finally, for entomopathogenic organisms, which may be considered as potentially hazardous to bees, usually linear or sigmoidal dose–response effects are not observed because mortality is due to pathogenicity and not due to toxicity. Therefore, at this time, it is not feasible to quantify different routes of exposure of microbial pesticides to bees; however, this represents an important area of new research in the future. On the other hand, further research regarding co-evolution dynamics between host and parasite and the host specificity at strain level can provide valuable information for development of microbial biopesticides.

## 5. ADDRESSING LIMITATIONS OF THE CURRENT TEST GUIDELINES

Most of the current test guidelines/guidance documents at the tier I level were developed to provide reliable and reproducible results regarding the effects of synthetic chemical pesticides on bees. Due to various differences in the properties of synthetic chemical pesticides compared to microbial biopesticides, some modifications of these test guidelines/guidance documents are needed. Therefore, we highlight needed modifications and provide suggestions which should be taken into account in the testing of microbial biopesticides on honey bees.

### 5.1. Observation period

Current adult honey bee study guidelines prescribe observation periods ranging from 48h for the OECD Guidelines No. 213/214 (OECD 1998a, b) acute oral/contact studies typically used for conventional chemical pesticides up to 30 days for the OCSPP 885.4380 tier I test design used for the assessment of microbial pesticides. At one end of this spectrum (i.e. 48h), observation periods are too short to detect adverse pathogenic effects from microbial test items, the majority of which exert their effects much more slowly than their chemical counterparts. However, study durations of 30 days are confounded by numerous methodological impediments to meeting the control mortality criterion of the test subjects. An optimal observation period should be determined that allows sufficient time to detect pathogenicity but does not cause method-related adverse effects or mortality on the bees themselves. The optimal observation period is likely longer than that required for studies with chemical pesticides, and it may vary depending on the microorganism and test species involved.

### 5.2. Dietary considerations

#### 5.2.1. Adult honey bees

Standard study designs for chemical testing cannot simply be extended to 30 days to make them appropriate for testing of microorganisms. In standard acute studies, adult bees can be taken directly from colonies. However, attaining the required lifespan and increasing the likelihood of meeting the control validity criterion in extended-duration studies requires the use of newly emerged worker bees, typically reared directly out of brood frames in a laboratory incubator. The life span of honey bees is dependent on protein and particular ratios of dietary essential amino acids (Paoli et al. 2014a, b). The standard 50% sucrose solution stipulated for acute toxicity studies in the OECD 213 and 214 guidelines is an inadequate long-term diet for juvenile honey bees, and high mortality can be expected unless the bees are provided with pollen or protein in their diet, the addition of which is known to increase longevity in honey bees (Archer et al. 2014; Di Pasquale et al. 2013). In addition, newly emerged honey bees do not consume sugar solution as readily as older honey bees (Jones et al. 2018; Paoli et al. 2014a) and therefore require an extended treatment period in oral studies or an acclimatisation period in order to consume 100–200 μL as stipulated in the OECD 213 and 214 guidelines.

Thus, pollen supplements or substitutes, as well as an acclimatisation period, should be considered for pathogenicity studies which require an extended observation period and the use of young, freshly emerged worker bees.

#### 5.2.2. Honey bee larvae

The OECD Guidance Document No. 239 assessing honey bee larval toxicity following repeated exposure was published under the responsibility of the Joint Meeting of the Chemicals Committee and the Working Party on Chemicals, Pesticides and Biotechnology in July 2016 (OECD 2016). Based on this guidance, individual larvae are exposed to the test chemical from day 3 to day 6 after eclosion, with the test item administered daily in an artificial diet containing royal jelly (50% w/w), as well as yeast extract, glucose and fructose in an aqueous solution. Mortality and any abnormal effects are recorded daily between day 4 and day 8 (i.e. larval mortality) and again on day 15 (i.e. pupal mortality). Finally, the rate of adult bee emergence in all treatment groups is assessed at the end of the study on day 22.

This test is best suited for the assessment of potential toxic effects of chemicals following repeated exposure. However, the assessment of pathogenicity is hampered both by the timing and route of exposure. In particular, royal jelly is known for its antimicrobial properties (Blum et al. 1959; Fujiwara et al. 1990; Fontana et al. 2004; Romanelli et al. 2011, Bilikova et al. 2015). An assessment of the inhibition potential of both royal jelly and artificial larval bee diet containing royal jelly on various microorganisms, including known bee pathogens, indicated growth suppression of all tested bacteria species (Schmehl et al. 2019).

Furthermore, the optimal timing of infection of larvae may precede the window of exposure (days 3 to 6) of the in vitro larval assay, reducing the rate of infection and the potential of this test to detect pathogenicity (Brødsgaard et al. 1998). Thus, further optimisation steps are needed to assess whether the current test design as stipulated by the OECD Guidance No. 239 represents an adequate assessment method for screening of microbial pathogenicity (OECD 2016).

### 5.3. Treatment groups

Studies are performed as “limit” tests that apply the microbial pesticide at a single rate but contain multiple groups (e.g. live culture; attenuated culture) in an effort to elucidate pathogenicity and toxicity; studies may also be performed using multiple rates so as to develop dose–response relationships. However, toxicity parameters generated from dose-response studies such as regression-based median lethal doses (LD_50_) and hypothesis-based no-observed adverse effect concentration (NOAEC) values may not be relevant to risk assessment schemes as trigger values indicating risk are typically calibrated for field exposure. However, field exposure to microbial pesticides cannot be as reliably estimated as for chemical-based pesticides.

Studies that attempt to distinguish pathogenicity from other effects such as toxicity or the physical attributes of the microbial pesticide typically include a live microbe group, an inactive microbe group or a sterile filtrate of the live microbe, and a blank control (USEPA 1996b). The use of inactive microbe and sterile filtrate treatment groups are attempts to distinguish pathogenicity from toxicity and the physical nature of the microbial pesticide. The OECD No. 67 stipulates that if metabolites are known to be responsible for the mode of action, toxicity data for the metabolites should be available. However, inactivating the microorganism is typically done by autoclaving, which can produce or denature toxins, and may denature metabolites and cause issues with homogenisation due to “clumping” of the test material. The ability to distinguish pathogenicity from toxicity or any physical effects of the microorganism is therefore limited (see Appendix).

The OCSPP 885.4380 states that the potential pathogenicity of the microbial test item should be assessed but gives no further guidance on how this might be achieved. Some laboratories have attempted to recover the test item from bees that died during the study. However, this is not a definitive test of pathogenicity and post-mortem saprophytic growth may be difficult to differentiate from any potential microbial pathogenicity.

### 5.4. Dose rates

The maximum hazard dose (MHD) is typically used in studies that test the microbial pesticide at a single rate. However, the use of the MHD may limit the screening ability of the tier I tests (i.e. tier progression would likely occur rather than tier I tests screening out unharmful microbial pesticides), if tests indicating adverse effects at the MHD are not followed by further testing at lower exposure levels. For example, for honey bee testing, the MHD can be defined as 100 × the maximum field application rate (OCSPP 885.4380). However, detrimental effects may be detected, but they may result from high-dose toxicity that might not occur at lower doses, thus masking pathogenicity. Testing should then take place at lower levels to better define the exposure level at which effects are observed. Testing at lower levels would also potentially reveal sublethal effects that may detrimentally impact bees, such as reduced flying or feeding.

The MHD has the unintended consequence of fixing dose with age and therefore limits results to effects on the individual. Eusociality and within-colony dynamics should be considered for potential pathogenicity. If the microorganism is infectious and replicates in the individual bee, then transmission to nest mates could occur at a dose higher than that applied in the laboratory.

### 5.5. Toxic reference

The use of a 24h LD50 from a chemical toxic reference substance outlined in the OECD 213 and 214 test guidelines has served as a measure of the study to detect a toxic treatment effect from acute oral and contact exposure. However, the endpoint is not reflective of the ability to resist infection and/or pathogenicity or the use of diet-supplemented newly emerged honey bees.

The necessity of using newly emerged bees that have been “health-optimised” by the addition of dietary pollen has an influence on their sensitivity to toxic reference substances. Diet, including pollen, is known to affect sensitivity to pesticides and the expression of genes relating to the detoxification of xenobiotic compounds. Nutritional status is also known to affect the ability of bees to resist disease (Foley et al. 2012; Dolezal and Toth 2018). Consequently, LD50 values obtained with healthy, well-nourished bees of similar age may extend beyond the published ranges for shorter-term studies that use mixed-age bees fed only on sugar solution.

Rather than develop new validity criteria for toxic reference items in long-term studies, consideration should be given to whether there is any value of such treatments in microbial studies. Toxic reference chemicals have little relevance to the evaluation of test items with microbial modes of action. Alternative approaches more suitable to testing with microbial pesticides may be needed to better ensure that the study is capable of detecting effects.

### 5.6. Environmental conditions

Current guidelines recommend that adult bees be maintained at 25 ± 2 °C (OECD 213 and 214) or 33 ± 2 °C (OECD 245; OECD 2017a) and at 50–60% relative humidity. Consideration also needs to be given to the growth conditions of the microbe (many of which require considerably higher relative humidity for at least the first few hours of the exposure period) while not exceeding the requirements of honey bees for longer-term studies. However, tailoring environmental conditions to the microorganism, rather than the test species, would likely be detrimental to honey bees, particularly at the larval stage.

### 5.7. Exposure duration

Exposure to the microbial pesticide varies amongst laboratories from the standard acute exposure period of 4–6h for chemical pesticides, to longer-term continuous exposure for oral studies. However, as discussed earlier in this overview, standard honey bee toxicity tests (acute and chronic) are not suitable for the assessment of potential pathogenicity, which would require greater focus on the observation window (i.e. study duration), rather than exposure duration. Consideration would need to be given to the stability of the microbe and requirements for dose verification with repeat dosing, typically performed by colony forming unit (CFU) counts on agar plates.

### 5.8. Data analysis

Any future method development should be guided by discussions of how the data can be analysed. For example, multiple group comparisons are required in order to distinguish between pathogenic effects and groups that control for toxicity and physical effects. Consideration should be given to appropriate statistical tests that account for changing mortality over the extended test period required for microbial pesticides.

## 6. FURTHER DISCUSSION POINTS

### 6.1. The potential for immune activation

Many microbial pesticides are unlikely to cause disease in non-target insects due to a lack of specific pathogenicity. However, simply exposing the insect to a microorganism has the potential to activate subclinical responses that can lead to colony-level effects. For example, injection with a non-pathogenic microbial cell surface complex induces a massive antimicrobial peptide response in bumblebees (*Bombus* spp.) and honey bees (Mallon et al. 2003; Laughton et al. 2011; Alaux et al. 2012; Siede et al. 2012). Furthermore, ingestion of a dietary cocktail of non-pathogenic bacteria has been demonstrated to increase transcription of an antimicrobial peptide gene in honey bee larvae (Evans and Lopez 2004). Immune activation alters many aspects of behaviour that may have adverse effects at both the individual and colony level. For example, immune activation increases transcription of genes related to foraging activity (Alaux et al. 2012) and leads to decreased queen attendance (Alaux et al. 2012), a reduction in learning (Mallon et al. 2003) and increased aggression toward immune active individuals (Richard et al. 2008). Increased aggression toward immune active individuals may translate into ejection from the colony, a mechanism that may be masked in the laboratory by the lack of interactions between treated and untreated individuals in the colony. The established paradigm uses laboratory trials as a “worst-case” scenario before progression to higher-tier field trials, which may mask the downstream immunological impacts on endpoints such as mortality.

### 6.2. The possible impacts of contaminants and adjuvants in the end use biopesticide product

Meikle et al. (2012) report microbial contamination of plant protection products. However, in many countries, controls are in place to prevent significant microbial contamination of plant protection products, though some background contamination can occur. For example, some contamination is expected in products containing insect viruses due to the nature of their necessary production within insect hosts. Nonetheless, regulations within several countries require identification and control of potential contaminants, including microorganisms (CFR 2021, EC 1107/2009a, EC 284/2013b; see also USEPA 1996c, d, e). While any testing during production is typically performed to ensure that human pathogens are not present, methods to prevent contamination by these microorganisms are generally understood to control the growth of other microbial contaminants (OECD 2011). Furthermore, additional information on toxicity, infectiveness and pathogenicity to bees of the plant protection product has to be reported, where it is not possible to predict the effects of the plant protection product on the basis of the data available for the microorganism (EC 284/2013b). It is not feasible to test all combinations of the microorganism with other formulation ingredients or other products that may or may not be included in tank mixes, which introduce some uncertainty into risk assessments depending on the extent to which they are used in tank mixes. However, additional testing with products in higher-tier semi-field and field studies can cover the testing of the adjuvants used in combination with such formulations where it is needed.

### 6.3. Additional approaches to improve testing and risk assessment

All of the issues described above should be addressed in any efforts to improve testing using current guidelines, revisions to current guidelines, or development of new guidelines. The ICP-PR Microbials and Bees Working Group presents these issues as discussion points for moving forward with these improvements and will continue to work through them. Below are additional approaches that may be taken to help improve testing and risk assessment for bees.

### 6.4. Testing with Apis or non-Apis bees

As with evaluating exposure, toxicity and risk to numerous taxa and recognizing that not all species that may come into contact with a plant protection product can be tested, regulatory authorities utilise data on surrogate species to represent these taxa. Surrogate species are selected based on multiple factors which include their commercial availability, ability to thrive under laboratory test conditions and the ease with which they can be manipulated. The Western honey bee (*Apis mellifera*) is used as representative (i.e. surrogate) species to evaluate the risk of plant protection products (including biopesticides) to all species of *Apis* and non-*Apis* bees, because they are readily available and are considered more suitable for use when assessing toxicity and exposure to bees (Hinarejos et al. 2019). Although studies (Arena and Sgolastra 2014; Thompson 2015) have indicated that data on honey bees are relatively protective for a broad range of *Apis* and non-*Apis* bees, concerns remain. There are more than 20,000 bee species worldwide (Michener 2007), including also bumblebees (*Bombus* spp), stingless bees (tribe *Meliponini*) and solitary bees, that may differ in their biology and ecological traits (e.g. sociality, flight or activity season, feeding, nesting materials, behaviour) compared to honey bees. There is uncertainty regarding the extent this variability and diversity may result in an increased pesticide sensitivity (infectivity and/or pathogenicity in the case of microbial pesticides) or exposure to non-*Apis* bees in comparison to honey bees (Vaughan et al. 2014). Joint efforts between academia, regulatory agencies and industry produced important advancements in understanding the extent to which exposure data for honey bees are protective for non-*Apis* bee exposure (Bireley et al. 2019; Boyle et al. 2019; Cham et al. 2019; Gradish et al. 2019; Hinarejos et al. 2019; Sgolastra et al. 2019).

There are currently no validated test protocols for most of these toxicity data points in non-*Apis* bees, and therefore, recommendations for non-*Apis* bees cannot yet be fulfilled. Protocols to evaluate the effects of conventional pesticides on bumblebees (*Bombus* spp.) and solitary bees (*Osmia* spp.) are being developed by the ICP-PR Bee Protection Group. As a result of these ICP-PR efforts, the OECD has already adopted standardised and validated test guidelines for acute oral and contact studies for bumblebees (OECD 246 & 247; OECD 2017b, c). Additional laboratory-based methods have been proposed as a means of investigating the effects of pesticides on bumblebees through the use of a queenless microcolony with a small flight cage (Mommaerts et al. 2012; Klinger et al. 2019). Nevertheless, such methods have disadvantages compared to queenright colonies, which represent conditions more realistic to the field (Van Oystaeyen et al. 2021).

Protocols to evaluate the acute effects of pesticides on solitary bees (*Osmia* spp.) in laboratory conditions are currently being developed. In this context, some recommendations for standardised oral toxicity test protocols for larvae of solitary bees, *Osmia* spp., were recently published (Eeraerts et al. 2020). However, similar technical challenges found with honey bee OECD protocols would apply to these new non-*Apis* bee studies (e.g. short exposure duration, route of exposure, need for more suitable toxicant reference and validity criteria). As with honey bees, efforts to improve the non-*Apis* bee testing with microbial pesticides using current guidelines, revisions to current guidelines or development of new guidelines would be applicable.

### 6.5. The (effect) modelling perspective on microbial pesticides

Honey bee colony simulation models have been identified as tools that could potentially be useful in pesticide risk assessments (EFSA 2016, 2021; Sponsler and Johnson 2017). While specific and appropriate test systems and protocols need to be developed for the quantification of effects of microorganisms on survival and reproduction of the relevant bee species, the modelling offers the opportunity to go beyond the specific experimental test conditions in the laboratory or in the field. Additionally, in comparison with field trials, modelling is likely to be economically efficient and could be more specifically applicable to varied localities (EFSA 2021).

Basically, three different domains of modelling can be differentiated in this context:

- Modelling of effects of microbial pesticides at the level of individual bees
- Modelling of effects of microbial pesticides at the colony/population or community levels
- Modelling of spread and persistence of the microorganisms

#### 6.5.1. Extrapolation of individual level effects

Modelling individual-level effects is possible (e.g. using an energy-based modelling approach). The DEBtox model is based on DEB (Dynamic Energy Budget) theory (Nisbet et al. 2004; Kooijman 2001), which captures the response of energy fluxes to changes of the organism and its environment. The model describes both toxicokinetics (quantification of metabolic and elimination processes) and toxicodynamics (toxic dose responses) which can provide understanding of time-related effects. While allowing calculation of standard LD_50_ values similar to probit analysis, DEB models provide significantly more powerful abilities to predict effects and describe toxicity dynamics. When linked with population dynamic processes, DEBtox could be used to model impacts on the life-span expectancy of individual bees, especially if the microbial pesticide is expected to accumulate over time or exhibit delayed toxicity (e.g. Hesketh et al. 2016).

Assuming that pathogen infections have an impact on energy use, the DEB theory could also be adapted to evaluate this effect (Kooijman 2001). A major challenge is that as the DEB theory was developed to account for individual-level growth and reproduction as a function of energy uptake and distribution. However, this is inconsistent with the life history of social bees where the hive-level reproduction is through a single bee (i.e. the queen). In addition, currently no adaptation of DEB models with microbial pesticides is available (i.e. the DEBtox models can only account for conventional (chemical) pesticides), and the species choice is restricted to *Apis* bees. These limitations represent exciting opportunities for future research, but they will also require substantial work.

Bee simulation models such as BEEHAVE (Becher et al. 2014) or the landscape-scale foraging model by Baveco et al. (2016) use a simple energy balance calculation, but they do not explicitly describe growth and reproduction of individual bees as do DEB models. Nevertheless, individual-level models for bees that account for survival, and probably also specific models for reproducing individuals within bee colonies or populations can be developed. These models could be parameterised to provide a means of evaluating the effects of microbial pesticides on survival and reproduction. Examples for such individual-level models for other non-target organism groups (e.g. aquatic insects) already exist for survival (GUTS; e.g. Jager and Zimmer 2012; Jager and Ashauer 2018) as they do as well for effects on growth and reproduction (e.g. Jager and Zimmer 2012). With such DEB model for bees, tests of microbial pesticides could be evaluated and the effects of exposure on survival and reproduction could be captured in model parameters and extrapolated to other untested conditions.

#### 6.5.2. Impact of effects on colony or population survival

Another challenge involves checking for possible consequences of effects on individual bees for the survival of the colony or population. Since toxic effects are interpreted as time-dependent parameters in DEBtox models, these models can be used to predict either short-term or long-term effects on key traits linked to population parameters (Kooijman 1993). The DEB models can therefore also be used to extrapolate toxic effects for single compounds measured at the individual level to meaningful consequences at the population level (Baas et al. 2010; Jager and Zimmer 2012).

Mechanistic models can also provide approaches to systematically compare interactions between toxicity, exposure and species (or taxon-)-specific trait combinations on population-level outcomes. Existing mechanistic modelling approaches can be used to check the consequences of a particular parameter on survival or reproduction (e.g. determining the level of forager mortality that affects the survival of the honey bee colony). Multiple honey bee colony simulation models have been introduced to improve understanding of the interplay of many processes and factors in honey bee colonies (Becher et al. 2013; Khoury et al. 2011; Becher et al. 2014; Betti et al. 2017; Kuan et al. 2018). The honey bee colony model BEEHAVE (Becher et al. 2014) was the first honey bee model to integrate processes both within the hive and in the landscape.

The European Food Safety Authority (EFSA) reviewed the BEEHAVE model with respect to its acceptability for use in risk assessment (EFSA 2015) and considered that its utility in a regulatory context is limited primarily because it lacks a complete pesticides exposure-effects module. A module to link exposure to pesticide residues in pollen to effects on the colony is available (Schmolke et al. 2019), but additional exposure routes including nectar, water and direct exposure of foragers, as well as sublethal effects, would need to be included. The extension of BEEHAVE with a complete pesticide exposure-effects module as well as efforts to develop another model to examine the population dynamics of bumblebees (Bumble-BEEHAVE; Becher et al. 2018) are currently underway according to the developer’s website (http://beehave-model.net/).

In 2016, EFSA completed a detailed technical report (EFSA 2016), outlining its vision for a mechanistic computer model for regulatory purposes to assist with risk assessment of pesticides in the context of multiple stressors and environmental factors on honey bee colony health. Based on their review, EFSA selected the agent-based simulation model ApisRAM, which is also supported by a parallel EFSA project on field data collection in different countries that will help to calibrate and validate the model in different EU landscapes (EFSA 2021).

The calibration and validation of any of these models from bee field research with microbial pesticides are of crucial importance, proving the model’s reliability for future regulatory applications. Structured laboratory studies and controlled field studies that attempt to examine the relationship between contaminant exposure and effects both play a role in model training, and may serve as better training sets than monitoring studies, which can be readily confounded by other variables, both known and unknown. As discussed earlier, there is no validated model available yet which simulates and predicts pesticide effects, but it will be essential in future developments that models can be parameterised with bee studies that have agreed upon protocols for microbial pesticides and with a transparent representation for how to link exposure in the landscape and the hive with the effects.

#### 6.5.3. Spread and persistence of microbial pesticides

A third potential application of modelling is the model-based assessment of pathogen spread (e.g. in a meta-population model or in a landscape) and the persistence of pathogens in infected bees or bee colonies/populations. Individual-based models (IBMs) are advantageous because individual bees are modelled in a spatially explicit environment, which could facilitate the simulation of the transmission of microorganisms as living, proliferating agents within a bee population. Such transmission dynamics could be simulated in the BEEHAVE model, or other landscape-scaled approaches (e.g. Baveco et al., 2016) could be used for this purpose. Such application of simulation models would be a good opportunity for the use of in silico models in cases where experimental work appears very challenging and resource-demanding. Parameters for persistence of microorganisms and transmission rates would need to be derived from laboratory experiments and could then be extrapolated to whole colony levels and environmental conditions by using simulation models. Simulation models can account for dynamic environmental conditions such as climate, habitat and additional stressors.

For any revision of test protocols, testing should be adapted to allow the parameterisation of modelling approaches. For tests on individual bee performance, for example, usually, observations of effects over time are needed to calibrate models. Other aspects include that experiments would focus on providing important model parameters; for microbial pesticides, this could, for example, be to test persistence of the microbe under relevant conditions and transmission rates between bees.

## 7. OVERALL CONCLUSIONS

The current suite of tests/tools for evaluating the effects of conventional pesticides has continued to evolve and now includes laboratory-based acute and chronic toxicity studies of individual *Apis* and non-*Apis* bees and semi-/full-field based studies of whole colonies. However, these tests have limited applicability for evaluating microbial pesticides. Since interest in and utility of microbial pesticides continue to increase, efforts are underway to enhance the suite of available tools with which researchers in academia and industry can evaluate bee exposure to and effects from the use of microbial pesticides. Of particular interest is developing standardised tests that regulatory authorities can use as lines of evidence in assessing the likelihood of adverse effects on bees from the use of microbial pesticides. While standardised test methods currently exist for evaluating microbial pesticides, the study conditions have not been optimised, presenting difficulty in obtaining reliable and consistent results.

This overview has identified both generic and specific challenges associated with testing of bees with microbial pesticides and has provided recommendations on opportunities to enhance testing methods and develop new approach methodologies. The overview has also identified the utility of simulation models that when appropriately parameterised could be used to extrapolate individual-based effects to colony and landscape-level impacts and reduce the need for testing. Overall, this overview provides a foundation with which regulatory authorities could identify/prioritise test development. While microbial pesticides have typically been considered less of a risk than conventional pesticides, there is a critical need to ensure that understanding is based on a strong foundation of science.

## Figures and Tables

**Table I:** Current available insect pollinator test guidelines

Test organism	Laboratory toxicity studies (tier 1)					Higher-tier studies	
	Adult			Larval		Brood	Colony
	Acute		Chronic	Acute	Chronic	Semi-field	Semi-field, field
	Contact	Oral	Oral	Single dose	Repeated dose		
Honey bees (*Apis mellifera*)	OECD 214/OCSPP 850.3020	OECD 213*	OECD 245	OECD 237	OECD GD 239	OECD GD 75/Feeding Oomen	EPPO 170/Large Colony Feedings Study/OCSPP 850.3040 (2010)
	Toxicity of residues on foliage OCSPP 850.3030						
Bumblebees (*Bombus* spp.)	OECD 246	OECD 247	NA	NA	NA	NA	NA
Solitary bees (e.g. *Osmia* spp.)	Draft OECD GL (being developed)	NA	NA	NA	NA	NA	NA

*NA* not available

* OCSPP Guideline 885.4380 provides information regarding testing of toxicity/pathogenicity via oral exposure in honey bees, but is neither specifically an acute nor chronic oral test

**Table II:** Summary of generic study design issues associated with current test guidelines and guidance documents used in assessing exposure and effects on bees

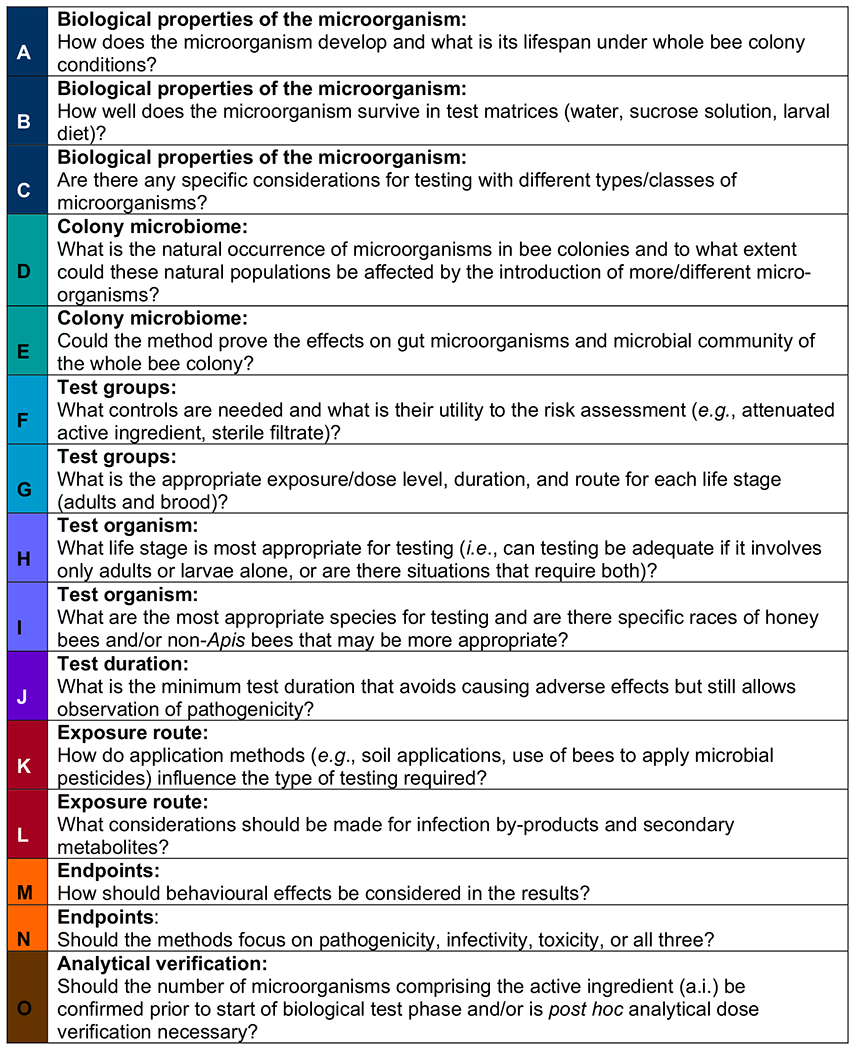

**Table III:** Specific limitations in the current bee test guidelines/guidance documents when testing microbial pesticides

Test	Study Type	Mode of Application/Test conditions and duration	Endpoints^1^	Fit for purpose	Specific Limitations	Generic Limitations^2^
**OCSPP 885.4380**	Honey bee adult oral toxicity	Oral exposure. No details given. “Testing in the hive may be necessary.” Duration ≥ 30 days	NOEC	Yes, with some modifications	Information on methods limited. As stand alone guidance not sufficiently informative. Need for suitable reference toxicant; need for suitable validity criteria.	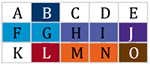
**OECD 213**	Honey bee adult acute oral toxicity	Single oral application, duration 48 to 96h. Test at 25°C	LD_50_	Yes, with some modifications	Study duration is too short and could be extended to >20 days when adapting bee age, diet, and test conditions.	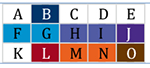
**OECD 214 / OCSPP 850.3020**	Honey bee adult acute contact toxicity	Single contact application, duration 48 to 96h. Test at 25°C	LD_50_	Yes, with some modifications	Study duration is too short and could be extended to >20 days when adapting bee age, diet, and test conditions. May not be most relevant exposure route.	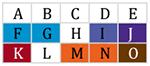
**OECD 237**	Honey bee larvae acute oral toxicity	Single oral exposure to larval stage, duration 7 days, endpoint is larval mortality, no pupal development is assessed.	NOEC	No	Single exposure may not be relevant for microbial testing.	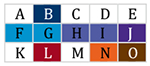
**OECD GD 239**	Honey bee larvae chronic oral toxicity	Chronic exposure to larval stage, duration 22 days, endpoint is hatching success.	NOEC	Yes, with some modifications	Larval exposure duration may need to be increased with first exposure timed at day 1, when larvae are most susceptible to infection, instead of day 3. Handling/assessment of dead larvae and pupae (pathogenicity signs/evidence, preparation and analysis of dead organisms) needs to be clarified. Endpoint should be NOEC.	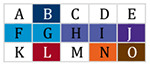
**OECD 245**	Honey bee adult chronic oral toxicity	Chronic oral exposure over 10 days, adapted test conditions to enhance longevity of caged bees	LDD_50_	Yes, with some modifications	Test design would need to be modified (supplemental pollen diet, modified caging system) to allow for a prolongation to at least 20 days, max. 30 days, while maintaining control mortality ≤ 20%.	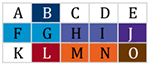
**OCSPP 850.3030**	Honey bee toxicity of residues on foliage	Acute contact test on treated alfalfa, duration 24h	RT_25_	No	Exposure duration may be too short or route may not be relevant for microbials in the most cases.	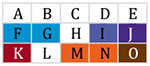
**OECD GD 75**	Brood semi-field	Spray application of bee attractive crop, semi-field test (tunnel or tent), direct exposure in tunnel 7 days, 19 days observation outside the tunnel	NOED	Yes, with some modifications	Oomen study (field, direct brood feeding) might be more suitable. Observation periods may need optimization.	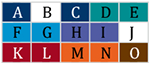
Oomen brood study (Oomen et al. 1992)	Field study, Brood feeding, repeated exposure	Oral exposure by feeding inside the hive in open field. Observation time not limited.	NOED	Yes, with some modifications	Test design applicable for microbials; however, the obtained endpoint may need to be made more relevant for microbial substances.	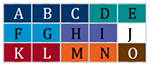
**EPPO 170**	Semi-field and field testing	Describes the conduct of trials for the evaluation of side-effects of plant protection products on honeybees, based upon the ‘*Recommendations for harmonization of methods for testing hazards of pesticides to honeybees.*’	NOED	Yes, with some modifications	Could be used as framework for general considerations in case field or semi-field studies are indicated. Observation periods may need optimization.	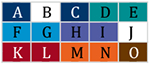
**OCSPP 850.3040**	Field testing for pollinators	No clear testing procedures described.	NOED	No	Referred to in the Guidance on Exposure and Effects Testing for Assessing Risks to Bees (USEPA 2016) in chapter 5.3 Microbial Pesticides. Not helpful as no specific information on testing is given.	-

1 NOEC = No Observed Effect Concentration; LD_50_ = Median Lethal Dose; LDD_50_ = Median Lethal Dietary Dose; RT_25_ = residual time required to reduce the activity of the test substance and bring bee mortality down to 25%; NOED = No Observed Effect Dose.

2 Generic limitations correspond to the colors and letters presented for generic study design issues in Table 2. If a box is colored, the limitation applies; white boxes indicate that the limitation does not apply.

## Data Availability

Not applicable.

## APPENDIX POSSIBLE CAUSES OF EFFECTS IN LIVE TEST ITEM GROUP

Possible causes:

Physical nature of Test Item (TI) × Toxicity* × Pathogenicity.

Physical nature of TI × Pathogenicity.

**Physical nature of TI × Toxicity***

Pathogenicity × Toxicity*

**Physical nature of TI** Pathogenicity.

Autoclave process may introduce confounding effects from formation of new toxins, denature toxins*, and alter the physical nature of the test item or cause “clumping”.

Effects in **bold** are those with no contribution from pathogenicity and are not controlled for due to potential confounds from autoclave process.

*Co-formulants + metabolites.
